# Natural History of Gastric Subepithelial Tumors: Long-Term Outcomes and Surveillance Strategies

**DOI:** 10.3390/jcm14186354

**Published:** 2025-09-09

**Authors:** Hye Kyung Jeon, Gwang Ha Kim

**Affiliations:** 1 Department of Internal Medicine, Pusan National University School of Medicine, Busan 49241, Republic of Korea; kyung3842@hanmail.net; 2 Biomedical Research Institute, Pusan National University Hospital, Busan 49241, Republic of Korea

**Keywords:** endoscopy, outcomes, stomach, subepithelial tumor, surveillance

## Abstract

Gastric subepithelial tumors (SETs) are commonly identified during routine endoscopy. Most SETs are asymptomatic and small (<2 cm) and exhibit benign behavior over time. Various histopathological types exist, including benign lesions, such as lipomas and heterotopic pancreas, and malignant lesions, such as gastrointestinal stromal tumors (GISTs). Endoscopic ultrasound (EUS) plays a critical role in evaluating the lesion size, layer of origin, border characteristics, and internal echogenicity. Approximately 4–15% of gastric SETs increase in size over ~5 years. The risk factors for the growth and malignant potential of SETs include initial tumor size, irregular or heterogeneous EUS features, mucosal ulceration, and confirmed GIST diagnosis. While lesions ≥2 cm in size or those with high-risk features are typically subjected to resection, small and low-risk SETs are managed with periodic EUS or endoscopic surveillance. Tissue acquisition via EUS-guided biopsy or endoscopic resection is warranted for indeterminate or suspicious cases. A risk-stratified approach minimizes unnecessary interventions while enabling timely detection of clinically significant lesions. Surveillance protocols should be tailored according to characteristics of SETs, patient comorbidities, and diagnostic confidence. This review highlights the long-term outcomes of gastric SETs, evaluates established risk factors for their growth and malignant potential, and discusses evidence-based strategies for surveillance and management.

## 1. Introduction

Gastric subepithelial tumors (SETs) present as smooth, elevated lesions covered by an intact mucosal surface on endoscopy. With the increased use of endoscopic screening, particularly in East Asia, gastric SETs are frequently incidentally detected in asymptomatic patients. They include a wide spectrum of histopathologies from benign lesions, such as lipomas, heterotopic pancreas, and leiomyomas, to malignant lesions, such as neuroendocrine tumors and gastrointestinal stromal tumors (GISTs). A key challenge in managing gastric SETs is distinguishing those that may progress or harbor malignancies from most indolent SETs. Conventional endoscopy alone may be insufficient to diagnose SETs due to limitations in sampling subepithelial tissue with forceps biopsy. Therefore, endoscopic ultrasound (EUS) has become an essential tool for characterizing SETs based on the layer of origin and internal echo features, thereby guiding the suspicion of a specific histopathology. Current clinical guidelines generally recommend periodic surveillance of small asymptomatic gastric SETs [1,2]. However, the natural history of these lesions, including their growth behavior and risk of malignant transformation, remains incompletely characterized. This review summarizes the long-term outcomes of gastric SETs, outlines known risk factors for growth or malignancy, and discusses evidence-based surveillance and management strategies.

## 2. Methods

We conducted a comprehensive literature search using the Web of Science, PubMed, and Google Scholar databases for articles addressing the natural history of gastric SETs indexed up to 31 May 2025. The following search terms were used: gastric subepithelial tumor, surveillance, and natural history of gastric subepithelial tumor. Studies published in languages other than English or without accessible full texts were excluded from the analysis. As a narrative review, this study is subject to inherent limitations, including the potential for publication and selection bias, as not all relevant studies may have been comprehensively captured. This study is a review of previously published literature and does not involve the collection of primary data or the participation of human subjects. Therefore, ethical approval and informed consent were not required.

## 3. Epidemiology of Gastric SETs

Gastric SETs are detected in approximately 0.7–1.7% of patients undergoing routine esophagogastroduodenoscopy, with increasing prevalence in older age groups [3,4]. In a Korean screening population, 1.9% of patients had gastric SET, which increased to 9.6% in those aged over 70 years [3]. Another Korean study reported a detection rate of 0.8% among more than 100,000 screening endoscopies [5]. The stomach is the most common site for upper gastrointestinal SETs [3,4]. Most gastric SETs are small (<2 cm), asymptomatic, and often discovered incidentally during endoscopy for unrelated indications or cancer screening.

Gastric SETs have various histopathological features. Mesenchymal tumors, such as leiomyomas and GISTs, are the most frequent, while other non-mesenchymal lesions include heterotopic pancreas, duplication cysts, and inflammatory fibroid polyps. GISTs are the most common and important SETs with malignant potential. Other SETs with malignant potential include neuroendocrine tumors and lymphomas. Overall, an estimated 60–80% of gastric SETs are benign lesions or very indolent neoplasms, while the remainder (mostly GISTs) have variable malignant potential [5].

EUS is the preferred diagnostic modality for the characterization of gastric SETs. EUS determines the layer of origin and sonographic features, such as size, border, and internal echogenicity. These specific features of EUS aid differential diagnosis; for example, a homogeneously hyperechoic lesion in the submucosa is likely to be a lipoma, whereas a heterogeneously hypoechoic lesion with cystic changes in the muscularis propria (MP) suggests a GIST [1]. It can help identify benign lesions (such as lipomas or cysts) without the need for invasive diagnostic procedures. However, in many cases, tissue diagnosis is still needed for a definitive diagnosis, especially when deciding whether resection is needed. Techniques for tissue acquisition include EUS-guided fine-needle biopsy (EUS-FNB), “bite-on-bite” deep forceps biopsy, unroofing or mucosal incision, and endoscopic submucosal dissection (for therapeutic and diagnostic tools) [6]. Each technique involves procedure-related risks, and diagnostic yields vary depending on the size and location of the lesion. Therefore, endoscopists must balance the need for diagnosis with the suspected benign or malignant nature and the size of the lesion with patient factors when deciding on invasive diagnostic procedures or recommending follow-up observations. In clinical practice, many small asymptomatic SETs (≤1 cm) without high-risk features are initially observed without tissue sampling.

## 4. Natural History of Gastric SETs

The progression of untreated gastric SETs remains incompletely understood. The primary clinical concerns are whether these tumors remain indolent, exhibit growth over time, or undergo malignant transformation. To date, limited information is available on this issue. Several recent large-scale studies and systematic reviews have provided valuable insights into this issue. Table 1 summarizes the key findings of representative studies on the natural history of gastric SETs. Most gastric SETs exhibit minimal or no growth during long-term follow-up. In a Korean cohort study of 130 patients with a mean follow-up period of 59.1 months, only 5.4% showed an increase in lesion size [7]. In a multicenter Korean cohort study involving 640 patients (mean follow-up duration of 47.3 months), the size increased in 4.2% of lesions [8]. Similarly, a Japanese multicenter study (NUTSHELL20 study), which included 824 cases, reported that small gastric SETs exhibited size progression in 8.5% of cases over a median follow-up period of 5 years [9]. Several additional studies from both Asian and Western populations have reported lesion enlargement in 5–15% of gastric SETs during follow-up periods ranging from 2 to 5 years (Table 1). Notably, studies focusing on very small lesions (<1 cm) reported low progression rates (<5%), whereas those that included slightly larger lesions or more tumors arising from the MP layer reported higher percentages of growth [10]. In addition, the heterogeneity in growth criteria among studies can affect the proportion of tumor growth: some studies defined “significant growth” with strict criteria (e.g., >25% increase and >5 mm absolute growth), while others used any measurable enlargement. Tumor growth generally progresses slowly over time. Song et al. noted that in 3.6% of 954 tumors, including 640 gastric SETs that increased ≥25% in diameter, the mean size increment was only 6.2 mm for 47.3 months [8]. In a systematic review of 41 studies, the growth rate of gastric SETs ranged from 0 to 2 mm/year in most cases [10]. Rapid or aggressive enlargement of the gastric SETs during surveillance is rare.

However, it is important to clarify that the absence of malignant transformation does not mean that no malignancies exist in published reports. Identifying the subset that progresses is crucial; hence, the risk factors are discussed in the next section.

## 5. Risk Factors for Growth and Malignant Potential of Gastric SETs

Although most gastric SETs remain indolent, a few increase in size or are found to have malignant potential on resection. Many studies have attempted to identify features that predict lesions that are likely to grow or harbor a malignancy. Risk factors can be categorized as lesion size, endoscopic/EUS features, and histopathological diagnosis. Table 2 summarizes the known risk factors and the supporting evidence.

### 5.1. Location of SETs

The anatomical location of SETs sometimes provides indirect information for predicting their malignant potential [6]. GISTs occur predominantly in the gastric body and fundus (upper two-thirds of the stomach), and an autopsy study found that 90% of microscopic gastric GISTs occur in the proximal stomach [17]. In contrast, SETs in the antrum are often heterotopic pancreatic or other benign lesions, such as lipomas and inflammatory fibrinoid polyps. Some studies have reported that SETs in the upper stomach are highly likely to be GISTs that exhibit growth, whereas SETs in the antrum rarely change [6]. However, location alone is not a strong independent histopathological predictor. Hu et al. reported that location (cardia/fundus vs. body vs. antrum) did not significantly differ between stable and growing SETs [13]. Nonetheless, endoscopists suggest that SETs in the antrum have a lower risk of growth and malignant potential, whereas those in the body or fundus, especially those arising from the MP layer, warrant greater attention due to the possibility of GISTs.

### 5.2. Initial Size of SETs

The most consistently reported predictor of tumor growth is the initial tumor size. Most studies have shown that SETs measuring 1–3 cm are more likely to enlarge over time than those measuring <1 cm [10]. Kim et al. reported that SETs 10–30 mm in size grew significantly faster (mean 0.22–0.31 mm/month) than those <10 mm in size (mean 0.14 mm/month) [12]. Song et al. also noted a positive correlation between initial lesion size and growth rate (*r* = 0.44, *p* = 0.009) [8]. Hu et al. reported an initially large tumor size as a predictor of tumor progression and identified 1.4 cm as the optimal cut-off tumor size associated with tumor progression [13]. Similarly, in a Japanese cohort, a cutoff of 13.5 mm was identified as the threshold above which the probability of growth markedly increased [9]. In addition, the initial size is a surrogate for the underlying histopathology; for example, almost all SETs >30 mm tend to be GISTs or other true neoplasms. Accordingly, current guidelines stratify management by size, with a more aggressive approach for larger lesions.

### 5.3. Mucosal Change Overlying SETs

The appearance of the overlying mucosa during endoscopy may provide clues regarding the malignant potential of SETs [6]. Intact normal-appearing mucosa is present in both benign and malignant SETs, whereas ulceration or erosion on the surface of SETs raises concerns regarding malignant SETs. Song et al. reported that SETs with mucosal changes (hyperemia, erosion, or ulcers) are highly likely to grow during follow-up (odds ratio [OR], 3.61) [8]. This may be because mucosal disruption may indicate tumor growth outpacing its blood supply or infiltration of the surface, which are classic surface features of malignant behavior. In clinical practice, the presence of a central ulcer or erosion on the surface of a gastric SET strongly suggests either a GIST or a neuroendocrine tumor and, less commonly, an SET-like carcinoma or lymphoma masquerading as an SET [6]. Such findings warrant prompt EUS and tissue sampling rather than routine surveillance.

### 5.4. EUS Findings of SETs

EUS enables detailed assessment of the shape and internal echogenicity of SETs. Several EUS features are associated with a high risk of growth or malignancy [20,21]. First, irregular lesion borders (ill-defined or lobulated margins) on EUS are important risk factors. Kim et al. reported that a lobulated shape was also found to be an important indicator of tumor growth with a higher OR than the other risk factors [15]; the proportion of high-risk GISTs was significantly higher in lobulated-shaped SETs compared to round-shaped SETs. In a 5-year study, SETs with irregular borders had 4.6-fold higher odds of significant growth than those with smooth, well-defined margins [3]. Similarly, Hu et al. reported that the progressive group had a significantly higher frequency of irregular borders than the stable group (40% vs. 11%, *p* = 0.002) [13]. Irregular borders may indicate an infiltrative growth pattern or a malignant histopathology. Another EUS feature is internal echogenicity; homogeneous hypoechoic lesions (common in leiomyomas) are less concerning than heterogeneous lesions with mixed echogenicity, cystic areas, or hyperechoic speckles [22]. Furthermore, cystic changes within SETs on EUS are another feature indicative of GISTs with a high risk of malignant potential [6]. The NUTSHELL20 study identified that internal cystic changes in EUS findings were significantly greater in the group with SET growth over time (*p* = 0.03) [9]. Cystic degeneration tends to occur in larger GISTs or those with rapid cell turnover/necrosis, suggesting a more aggressive biology. Other EUS features that raise suspicion of malignancy include deep ulceration and regional lymph node enlargement, although these are less commonly observed in small SETs. Taken together, EUS characteristics can stratify risk. For example, a 15 mm-sized SET in the MP layer, which is homogenously hypoechoic with smooth margins, is likely a leiomyoma with a low risk of growth, whereas a 15 mm-sized SET that is lobulated with mixed echogenic foci or cystic areas is more likely a GIST that can enlarge or already have malignant potential [1].

### 5.5. Histopathology of SETs

Ultimately, SET histopathology is the strongest determinant of malignant potential. Histopathology influences growth: benign mesenchymal tumors, such as leiomyomas and schwannomas, tend not to grow during surveillance, whereas GISTs have a greater chance of increasing in size (although slowly in several cases). A confirmed benign lesion (e.g., lipoma, duplication cyst, inflammatory fibroid polyp, or leiomyoma) has nearly zero risk of malignancy. Accordingly, if such a diagnosis is made, often via EUS-FNB or resection, further surveillance is not needed [1]. However, if an SET is diagnosed as a GIST, its malignant potential should be assessed, even if it is small.

Many of these risk factors are interrelated (e.g., a larger lesion is more likely to be a GIST, and a GIST is more likely to have irregular EUS features). Multivariate analyses have reported varying results on the initial size, mucosal ulceration, and EUS features as independent predictors of growth [3,8]. By recognizing these high-risk features, endoscopists can determine how to approach gastric SETs. For example, a 12 mm-sized SET with regular borders and no worrisome signs can be safely observed, whereas a 12 mm-sized SET with ulcers or irregular borders might prompt biopsy or resection.

## 6. SETs Located at the MP Layer

Gastric SETs arising from the MP layer have received special attention as this category includes the most clinically significant lesions, notably GISTs, as well as some benign tumors (leiomyomas and schwannomas) that can mimic GISTs. The management strategy is often decided based on whether an SET is suspected to be a GIST or a benign entity.

### 6.1. GISTs

GISTs are the most common mesenchymal neoplasms of the stomach and account for a large proportion of gastric SETs that eventually become neoplastic. They originate from the interstitial cells of Cajal in the MP and are characterized by mutations in KIT and PDGFRA. All GISTs have malignant potential. The prognosis and management of GISTs are guided by tumor size, mitotic index, and location. The Fletcher NIH criteria and subsequent risk schemes stratify GISTs into very low, low, intermediate, or high risk of recurrence or metastasis based on size and mitoses [18,19,23]. For example, a gastric GIST ≤ 2 cm with ≤5 mitoses/50 high-power fields is classified as very low-risk (0–3% chance of metastasis), whereas a tumor > 5 cm or with high mitotic counts is high-risk (significant metastatic potential). Most small gastric GISTs (<2 cm) grow slowly, and the risk of metastasis during surveillance is remarkably low [19]. However, in the presence of high-risk features by imaging such as irregular borders, cystic spaces, ulceration, echogenic foci, or heterogeneity, resection is recommended [24]. The rate of metastatic spread increases with the size of the lesion and may be as high as 86% for lesions greater than 10 cm with a high mitotic rate [25].

Given the malignant potential of GISTs, the current guidelines recommend definitive treatment for those ≥2 cm in size or showing high-risk features, and individualized management for smaller GISTs without high-risk features by imaging, such as irregular borders, cystic spaces, ulceration, echogenic foci, or heterogeneity. From a natural history standpoint, in virtually all series of gastric SETs, whenever lesions grew or were resected for suspicion, those that turned out to be malignant were GISTs; no other gastric subepithelial pathology (e.g., ectopic pancreas or leiomyoma) became malignant over time [11,12]. This reinforces the need to distinguish between GISTs and non-GISTs as gastric SETs.

### 6.2. Differential Diagnosis with GISTs

Leiomyomas are benign smooth muscle tumors that can arise from the MP, most commonly in the esophagus and stomach, particularly in the cardiac region. Gastric leiomyomas are benign, with no malignant transformation into leiomyosarcomas. A typical leiomyoma is a small (<3 cm) round homogeneous lesion often found near the gastroesophageal junction [6,22]. The natural history of gastric leiomyomas is essentially no or extremely slow growth. Park et al. reported that in 84 gastric leiomyomas followed up for 50.8 months, the tumor size increased in only two cases (2.4%) [26]. Hu et al. reported four gastric leiomyomas followed by EUS, and none showed any size increase over a median period of 1.5 years [13]. Other studies showed similar results in that SETs later identified as leiomyomas were invariably stable on serial endoscopy [10]. The primary challenge with leiomyomas lies in their diagnosis, as they closely resemble GISTs on imaging. Features favoring leiomyomas include their location in the cardia or within the esophageal lumen and EUS findings of a uniformly hypoechoic lesion contiguous with the MP. If EUS-FNB confirms leiomyoma, no intervention is necessary unless the tumor size increases or becomes symptomatic. Large symptomatic leiomyomas can be removed to relieve the symptoms; however, this is rarely necessary.

The other two mesenchymal tumors arising from the MP are schwannomas and glomus tumors. Gastric schwannomas are benign nerve sheath tumors that comprise a small percentage of gastric SETs. They are usually 2–5 cm well-demarcated masses, often with a distinctive peripheral lymphoid cuff on histopathological examination [27]. Schwannomas are essentially benign, and no malignant behavior is expected. Surveillance is not required in cases diagnosed using EUS-FNB or resection biopsy. Glomus tumors of the stomach are rare mesenchymal tumors that are typically benign; however, only a few malignant cases have been reported. They often appear as SETs in the antrum [28]. Glomus tumors are considerably less common than GISTs. When encountered, they tend to remain localized and indolent.

Differentiating between these lesions on EUS can be challenging, and various scoring systems or algorithms have been proposed (e.g., based on tumor shape, border, echogenicity, and patient age) [6,22,28]. In practice, if a lesion is strongly suspected to be benign, it can be safely observed. If features suggest GISTs, most guidelines advise at least one attempt at tissue diagnosis or removal.

## 7. Management and Surveillance Strategies

In this section, we summarize the current surveillance strategies and treatment indications for gastric SETs based on guidelines from the American College of Gastroenterology (ACG) 2023, European Society of Gastrointestinal Endoscopy (ESGE) 2022, and the American Gastroenterology Association (AGA) 2022 [29] (Table 3).

All guidelines unanimously recommend that gastric SETs with symptoms or high-risk features should be considered for removal rather than surveillance. If an SET causes bleeding, obstruction, or significant pain, resection is usually indicated, regardless of the histopathological findings [2]. However, if an asymptomatic SET is highly suspected to be benign (e.g., an incidental 5 mm lipoma or ectopic pancreas identified on EUS), guidelines state that no surveillance is necessary [1].

Generally, a 2 cm size threshold has been used as a rule of thumb: SETs ≥ 2 cm are often recommended for resection due to an increased malignancy risk, whereas those <2 cm are candidates for surveillance [1,2]. The ACG guidelines suggest resection for all gastric SETs that are confirmed as GISTs or suspected GISTs > 2 cm, as well as for GISTs of any size in non-gastric locations (since intestinal GISTs are more aggressive) [2]. For gastric GISTs <2 cm without high-risk features, the ACG guidelines noted insufficient evidence to mandate resection in every case. Therefore, they support surveillance or resection based on patient preference, and if the clinical decision is to resect, endoscopic methods may be considered as acceptable alternative therapies compared with surgery. ESGE guidelines recommend that any SETs ≥ 20 mm be diagnosed via EUS-FNB and, if it is proven GIST, recommends resection [1]. For smaller SETs < 20 mm, GISTs, or unknown histology, after the failure of attempts to obtain a diagnosis, the ESGE guidelines suggest endoscopic resection as an alternative to surveillance to avoid unnecessary follow-up. The Japanese GIST guidelines also indicate that surgery is the standard treatment for operable GISTs > 2 cm, whereas regular surveillance can be considered for those <2 cm without high-risk EUS features [30].

For lesions selected for surveillance, the ESGE guidelines provide a detailed interval schedule based on size [1]. If gastric SETs are <10 mm in size and asymptomatic with no definite diagnosis, an initial follow-up endoscopy at 3–6 months is recommended, followed by subsequent surveillance every 2–3 years. The rationale is to confirm no or slow growth early (at 6 months) and then perform surveillance, given the low growth rates of such lesions. If gastric SETs are 10–20 mm in size and not resected, ESGE guidelines suggest endoscopy at 3–6 months, followed by endoscopy at 1–2 year intervals thereafter, if there is no change. The short follow-up interval reflects a high, although small, risk of growth within this size range. If asymptomatic SETs >20 mm in size are not resected (e.g., patients unfit for surgery or refusal), the ESGE guidelines suggest close follow-up: endoscopy plus EUS at 6 months, followed by at 6–12 month intervals if still under surveillance. In practice, however, almost all gastric SETs > 2 cm (especially if GISTs are likely) are referred for removal, given their increased risk of malignant potential.

The above-mentioned surveillance recommendations are based on very low-quality evidence but represent a consensus that aims to catch any changes promptly. The ACG 2023 guidelines do not prescribe specific time intervals owing to the lack of high-quality data [2] but suggest that lifelong surveillance may be needed for unresected lesions and that some patients might prefer resection to avoid the burden of indefinite endoscopies.

Advances in endoscopic resection techniques now allow many gastric SETs to be removed endoscopically, particularly those <3–4 cm in size arising from the MP [31,32]. The ESGE guidelines suggest that for proven small GISTs (<20 mm), endoscopic resection by an expert can be considered an alternative to surgery after a multidisciplinary discussion [1]. Endoscopic submucosal dissection and endoscopic full-thickness resection have shown high *en bloc* resection rates of GISTs up to 30 mm with low recurrence [33,34,35]. However, these procedures carry some risks (perforation, which might require surgical rescue) and should only be performed in experienced centers. Minimally invasive surgery (laparoscopic wedge resection) remains the standard procedure for large tumors and tumors in difficult locations. From a surveillance perspective, if endoscopic resection is complete and histopathology confirms a benign or low-risk lesion, no further surveillance is required. If the resection margins are positive or indeterminate for a neoplasm, guidelines advise follow-up endoscopy at 3–6 months to evaluate the residual tumor. Recently, endoscopic resection has provided a middle ground rather than indefinite surveillance, and intermediate-risk lesions can be removed with minimally invasive procedures.

Notably, these recommendations are based on low-level evidence and may be reviewed as more prospective data become available. The central issue is risk stratification, followed by a tailored follow-up based on that risk. This approach ensures that patients with truly benign lesions are not overburdened with the procedures, whereas those with a potential malignant risk (such as small GISTs) are not lost to follow-up until too late.

## 8. Conclusions

**Figure 1:**
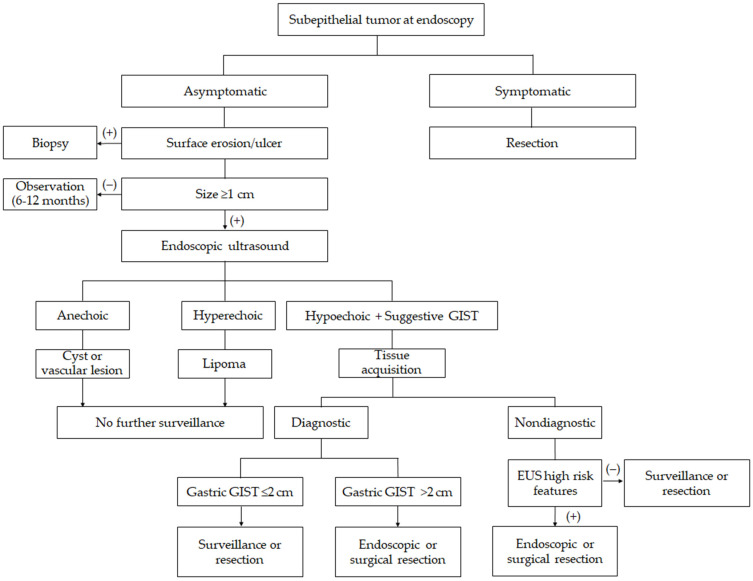
Suggested flowchart for managing gastric subepithelial tumors.

In clinical practice, the management of gastric SETs requires an individualized approach (Figure 1). Factors, such as patient age, comorbidities, anxiety, and access to expert endoscopists, must be weighed along with tumor characteristics. A patient-centric approach that discusses surveillance versus resection options in the context of the data is recommended. Current evidence suggests a conventional non-interventional approach for small gastric SETs. Long-term prospective registries are warranted to refine risk stratification models for the optimal surveillance and management of gastric SETs. Evidence supports adopting endoscopic surveillance for most incidental gastric SETs < 2 cm in size, reserving intervention for those that demonstrate growth or possess high-risk features, or for any confirmed GIST ≥ 2 cm. This strategy ensures patient safety while avoiding unnecessary procedures, in line with the typically indolent behavior of most incidental gastric SETs.

## Figures and Tables

**Table 1:** Summary of published reports on the natural history of gastric subepithelial tumors.

Study (Year)	No. Lesions	Population /Design	Follow-Up Period	Size Increase			Malignancy Yield
				Definition of Growth	Results	Increase Relative to Initial Size	
Bruno et al. (2009) [11]	49	SETs from MP <3 cm/ prospective	31.0 months (mean)	≥25% increase	5/49 (10.2%)	NA	0% overt malignancy; 3 of 4 resected lesions were GISTs (all very-low- and low-risk); 1 glomus tumor
Lim et al. (2010) [7]	130	SETs/ retrospective	59.1 months (mean)	≥25% and ≥5 mm	7/130 (5.4%)	NA	2 of 3 resected lesions ≥ 3 cm were GISTs (intermediate- and high-risk); 1 schwannoma
Kim et al. (2011) [12]	989	SETs ≤30 mm/ retrospective	24 months (median)	≥25% increase	84/989 (8.5%)	<10 mm: 29/450 (6.4%) 10–20 mm: 38/366 (10.4%) 20–30 mm: 17/173 (9.8%)	Among 25 resected lesions for changes, 19 were GISTs (3 high-risk, 4 intermediate-risk)
Song et al. (2015) [8]	640	SETs/ retrospective	47.3 months (mean)	≥25% increase	27/640 (4.2%)	NA	0% clinical malignancy during follow-up; 2 of 3 resected lesions were GISTs (low- or intermediate-risk)
Hu et al. (2017) [13]	88	SETs 1–3 cm from MP/retrospective	24.6 (stationary subgroup)/30.7 (progressive subgroup) months (mean)	≥20% increase	25/88 (28.4%)	NA	Among 17 surgically resected cases, 13 were GISTs (1 high-risk, 2 intermediate-risk)
Ye et al. (2020) [14]	410	SETs ≤20 mm/ retrospective	28 months (median)	Not specified	8/410 (2.0%)	<10 mm: 2/291 (0.7%) 10–20 mm: 6/119 (5.0%)	5 of 6 resected lesions due to increased size were GISTs (2 intermediate-risk, 3 low-risk)
Kim et al. (2022) [15]	1859	SETs/ retrospective	59.4 months (mean)	≥25% increase	138/1859 (7.4%)	<10 mm: 34/840 (4.1%) 10–20 mm: 59/673 (8.8%) >20 mm: 34/346 (13.0%)	50 of the 73 resected lesions were GISTs (4 high-risk showed increased size)
Abe et al. (2023) [9]	824	SETs ≤20 mm/ multicenter retrospective	5 years (median)	≥20% increase	70/824 (8.5%)	1–5 mm: 16/298 (5.4%) 6–10 mm: 23/344 (6.7%) 11–15 mm: 20/112 (17.9%) 15–20 mm: 11/70 (15.7%)	26 of the 34 resected lesions were GISTs (1 high-risk)
Choe et al. (2023) [4]	135	SETs ≥1cm/ multicenter retrospective	52 months (median)	Not specified	20/135 (14.8%)	10–20 mm: 13/113 (11.5%) >20 mm: 7/22 (31.8%)	Among 20 resected cases, 12 were GISTs and 1 was lymphoepithelioma-like carcinoma
Heo et al. (2024) [3]	262	SETs/ retrospective	58 months (median)	>25% increase and >5 mm	22/262 (8.4%)	NA	0% developed overt malignancy. 2 of 7 resected lesions were GISTs (1 intermediate-risk, 1 low-risk)
Iwamuro et al. (2024) [16]	610	SETs ≤20 mm/ prospective multicenter	4.6 years (mean)	≥5 mm increase	32/610 (5.7%)	NA	25 of 30 resected lesions were GISTs

SETs, subepithelial tumors; MP, muscularis propria; EUS, endoscopic ultrasound; GIST, gastrointestinal stromal tumor; NA, not available.

**Table 2:** Risk factors associated with growth or malignancy in gastric subepithelial tumors under surveillance.

Risk Factors	Supporting Evidence
*Endoscopic findings*	
Proximal location (fundus/body)	90% of microscopic GISTs were found in the fundus/body [17] Mid-third gastric location (OR: 1.65, 95% CI: 1.08–2.52) [15] Antrum SETs are frequently benign (pancreatic rests) with minimal growth [6]
Larger initial size	Lesions 10–30 mm grew faster than <10 mm lesions [12] Progressive group had initially larger size (*p* = 0.020) [13] Growth rates correlate with baseline size (*r* = 0.44) [8] Size ≥13.5 mm predicted growth in small SETs [9] GIST risk classification is size-dependent (metastatic risk rises sharply >5 cm) [18,19] Large initial tumor size (OR: 1.03, 95% CI: 1.01–1.05) [15]
Overlying mucosal changes (ulceration, erosion, redness)	3.6-fold higher odds of growth if mucosal ulcer/erosion present [8] Surface ulcer or erosion (OR: 2.47, 95% CI: 1.21–5.06) [15]
*EUS findings*	
Irregular border	Irregular/lobulated margins conferred OR 4.6 for significant enlargement [3] Progressing lesions had more irregular borders than stable lesions (40% vs. 11%) [13]
Heterogeneous internal echo	Uniformly homogenous hypoechoic lesions rarely enlarged [13] Heterogeneous echotexture was more frequent in lesions that grew [13] Mixed echogenicity/cystic areas were linked to GISTs that increased in size [9]
Internal cystic spaces	Presence of cystic change was a significant risk factor for growth in small GISTs [9] Cystic areas were often seen in large or high-risk GISTs (reflecting necrosis) [6]
*Histopathology*	
GISTs (versus benign lesions)	In univariate analysis, GISTs had more frequent size increase than other tumor types [12] All lesions that progressed to require resection were ultimately GISTs in several series [10,11] Leiomyomas, lipomas, etc., rarely grew or turned malignant [13] High-risk features (mitotic count, size >5 cm, etc.) in GISTs correlate with an 80% chance of metastasis (whereas small low-mitotic GISTs with 2–3% risk) [19]

GIST, gastrointestinal stromal tumor; SETs, subepithelial tumors; OR, odds ratio.

**Table 3:** Comparisons of guidelines for gastric subepithelial tumors.

Category	ACG (American College of Gastroenterology)	ESGE (European Society of Gastrointestinal Endoscopy)	AGA (American Gastroenterological Association)
Initial evaluation	EUS is suggested preferentially over endoscopy or contrast-enhanced cross-sectional imaging.	EUS is recommended as the best tool to characterize SET features.	EUS is the modality of choice for indeterminate SET when biopsies are nondiagnostic.
Tissue acquisition	EUS with tissue acquisition is suggested to improve diagnostic accuracy. EUS-FNB alone or EUS-FNA with ROSE; unroofing or tunnel biopsy if nondiagnostic.	Tissue diagnosis for SETs with features suggestive of GIST, if they are of size >20 mm, or have high risk stigmata, or require surgical resection or oncological treatment SETs < 20 mm: MIAB (first choice) or EUS-FNB (second choice) SETs ≥ 20 mm: EUS-FNB or MIAB equally effective	SETs arising from the submucosa: using tunnel biopsy (or deep-well biopsies), EUS-FNA, EUS-FNB, or advanced endoscopic techniques (unroofing or endoscopic submucosal resection) SETs arising from MP: EUS-FNB or EUS-FNA to determine whether the lesion is a GIST or leiomyoma
Benign lesions	No specific details provided	ESGE recommends against surveillance of asymptomatic gastrointestinal leiomyomas, lipomas, heterotopic pancreas, granular cell tumors, schwannomas, and glomus tumors, if the diagnosis is clear.	SETs that have an endoscopic appearance consistent with lipomas, pancreatic rests, and duplication cysts do not need further evaluation or surveillance.
Asymptomatic lesions without a definite diagnosis	There is insufficient evidence to make definitive recommendations regarding surveillance intervals when resection is not undertaken.	ESGE suggests surveillance of asymptomatic gastric SETs without definite diagnosis, with EGD at 3–6 months; <10 mm lesions every 2–3 years and 10–20 mm lesions every 1–2 years. >20 mm (if not resected): EGD+EUS at 6 months and then at 6–12 month intervals. SETs < 20 mm and of unknown histology after failure of attempts to obtain a diagnosis: endoscopic resection is an option to avoid unnecessary follow-up	SETs arising from MP < 2 cm: surveillance using EUS; otherwise tailored by features.
Symptomatic or ulcerated bleeding lesions	All guidelines recommend resection regardless of the size of the lesion and without a pre-resection diagnosis.		
Gastric GIST < 2 cm	There is insufficient evidence to recommend surveillance vs. resection	Surveillance or resection is both acceptable; resection considered after multidisciplinary discussion.	Can be surveilled using EUS
Gastric GIST ≥ 2 cm	Resection	Resection	Resection

EUS, endoscopic ultrasound; SET, subepithelial tumor; GIST, gastrointestinal stromal tumor; FNA, fine-needle aspiration; FNB, fine-needle biopsy; MIAB, mucosal incision-assisted biopsy; ROSE, rapid on-site evaluation; MP, muscularis propria; EGD, esophagogastroduodenoscopy.

## Data Availability

No new data were created or analyzed in this study.

## Abbreviations

The following abbreviations are used in this manuscript:

ACG	American College of Gastroenterology
ESGE	European Society of Gastrointestinal Endoscopy
AGA	American Gastroenterology Association
EUS	Endoscopic ultrasound
GIST	Gastrointestinal stromal tumor
MP	Muscularis propria
SET	Subepithelial tumor
FNA	Fine-needle aspiration
FNB	Fine-needle biopsy
MIAB	Mucosal incision-assisted biopsy
ROSE	Rapid on-site evaluation
OR	Odds ratio
